# Engaging Refugees With a Culturally Adapted Digital Intervention to Improve Sleep: A Randomized Controlled Pilot Trial

**DOI:** 10.3389/fpsyt.2022.832196

**Published:** 2022-02-23

**Authors:** Kerstin Spanhel, Eva Hovestadt, Dirk Lehr, Kai Spiegelhalder, Harald Baumeister, Juergen Bengel, Lasse B. Sander

**Affiliations:** ^1^Department of Rehabilitation Psychology and Psychotherapy, Institute of Psychology, University of Freiburg, Freiburg, Germany; ^2^Department of Health Psychology, Institute of Psychology, Leuphana University Lueneburg, Lueneburg, Germany; ^3^Department of Psychiatry and Psychotherapy, Faculty of Medicine, Medical Center–University of Freiburg, Freiburg, Germany; ^4^Department of Clinical Psychology and Psychotherapy, Institute of Psychology and Education, Ulm University, Ulm, Germany

**Keywords:** culturally sensitive treatment, refugees, healthcare barriers, sleep disturbances, transdiagnostic symptoms, internet-based interventions, low-threshold treatment

## Abstract

Refugees are exposed to multiple stressors affecting their mental health. Given various barriers to mental healthcare in the arrival countries, innovative healthcare solutions are needed. One such solution could be to offer low-threshold treatments, for example by culturally adapting treatments, providing them in a scalable format, and addressing transdiagnostic symptoms. This pilot trial examined the feasibility, acceptance, and preliminary effectiveness of a culturally adapted digital sleep intervention for refugees. Sixty-six refugees participated, with 68.2% of them seeking psychological help for the first time. Only three participants did not show clinically significant insomnia severity, 93.9% reported past traumatic experiences. Participants were randomly assigned to the intervention group (IG) or the waitlist control group (CG). Insomnia severity, measured by the Insomnia Severity Index, and secondary outcomes (sleep quality, fear of sleep, fatigue, depression, wellbeing, mental health literacy) were assessed at baseline, 1 and 3 months after randomization. The self-help intervention included four modules on sleep hygiene, rumination, and information on mental health conditions associated with sleep disturbances. 66.7% of the IG completed all modules. Satisfaction with the intervention and its perceived cultural appropriateness were high. Linear multilevel analyses revealed a small, non-significant intervention effect on insomnia severity of Hedge's g = 0.28 at 3-months follow-up, comparing the IG to the CG [*F*_2, 60_ = 0.88, *p* = 0.421]. This non-confirmatory pilot trial suggests that low-threshold, viable access to mental healthcare can be offered to multiple burdened refugees by culturally adapting an intervention, providing it in a scalable format, and addressing a transdiagnostic symptom.

## Introduction

The United High Commissioner for Refugees (UNHCR) reports on 82.4 million forcibly displaced people in the end of 2020, including 34.4 million people displaced abroad^1^,^1^ (1). This poses challenges on healthcare systems in the arrival countries (2–5), mainly because refugees are exposed to many stressors before, during, and after flight (6, 7). The many stressors influence refugee's mental health (8–10) and, correspondingly, the prevalence of trauma-associated disorders such as posttraumatic stress disorder or depression is enhanced (11–14). However, there is a mental health treatment gap (15): Individual barriers (e.g., language barriers, somatic concept of mental health, stigmatization) (16, 17) and structural barriers (e.g., lack of treatment resources or financing opportunities) (18, 19) hinder refugees from seeking help for mental disorders. To overcome mental healthcare barriers, various approaches have been proposed (20, 21), among them (a) using scalable and low-threshold treatment formats, (b) addressing somatically perceived and transdiagnostic symptoms, and (c) culturally adapting treatments.

Concerning (a) the use of scalable and low-threshold treatment formats (22–24), digital interventions are suggested to be easier to access for refugees than face-to-face treatments (25–27), as they are anonymous, resource saving, and flexible in use regarding time and place (28–30). With numerous evidence of efficacy in western populations (31–33), findings on the efficacy of digital interventions among refugees are inconsistent: Whereas a digital intervention to reduce posttraumatic stress disorder did not yield a significant effect in a randomized controlled trial (34), results of two conducted randomized controlled pilot trials indicated significant effects of the digital interventions in reducing depressive symptoms (35, 36). (b) Addressing somatically perceived and transdiagnostic symptoms might be another option to overcome barriers among refugees (37–39). Mental distress is often linked with somatic symptoms in refugee's countries of origin (40), and somatic mental health concepts are prevalent (41, 42). Sleep disturbances as a somatically perceived mental health problem are frequently reported (43–45), which suggests that they could be an accepted way to express mental distress among refugees. Moreover, sleep disturbances are a transdiagnostic symptom (46, 47) and, herewith, often associated with other mental health conditions among refugees (48, 49). Indeed, treating sleep disturbances–also via digital interventions–has been shown to reduce associated mental disorders such as depression and posttraumatic stress disorder (50–54). To date, only two studies evaluated effects of interventions on sleep disturbances, with one study showing a positive effect of relaxation music on sleep quality in refugees (55), and a pilot trial indicating an improved insomnia severity following a transdiagnostic digital intervention (35).

Barriers could be further reduced by (c) culturally adapting treatments, that is by considering refugees' contexts and strains in their treatment (56, 57). Thereby, it appears relevant to adapt interventions in a systematic way (58), which is enabled by frameworks of culturally adapting face-to-face treatments (59–61) and scalable and low-threshold interventions (62–64). Culturally adapting face-to-face treatments seems to enhance their acceptance and efficacy among populations that differ from the original target groups (65, 66). Similarly, culturally adapted digital interventions are shown to be effective in reducing mental disorders (64, 67). However, only few digital interventions have been culturally adapted for refugees (68–71), with the efficacy of some interventions being indicated in randomized controlled pilot trials (35, 36).

Combining the three illustrated approaches to overcome mental healthcare barriers among refugees, we culturally adapted a digital sleep intervention. A randomized controlled pilot trial was conducted to evaluate the feasibility, acceptance, and preliminary effectiveness of the resulting eSano Sleep-e intervention among refugees, which should allow drawing conclusions on conducting a final randomized controlled trial. Thereby, we aimed to investigate

1. whether refugees adhered to the intervention;
2. whether refugees were satisfied with the intervention and perceived it as culturally appropriate; and
3. whether preliminary evaluations indicate that the intervention improved insomnia severity and other sleep associated and mental health conditions in refugees.

## Materials and Methods

### Study Design, Participants, and Procedure

This is a randomized controlled pilot trial with an intervention group (IG) receiving access to the eSano Sleep-e intervention and a waitlist control group (CG). The study was registered in the German Clinical Trial Register (identifier: DRKS00018949). Deviating from the registration protocol, more than the 40 initially planned participants were included [the planned number based on the Consolidated Standards of Reporting Trials (CONSORT) guidelines for pilot and feasibility studies (72) and an assumed dropout rate of 20% (73)], due to (a) adjustments in the study procedure after the first enrolments of participants and (b) a high response following a last recruitment wave. Furthermore, recruitment was expanded from personal recruitment in Freiburg, Germany, to an online recruitment all over German-speaking countries, due to the restrictions in contacting people in person resulting from the Covid-19 pandemic. The study was approved by the commissioner for data protection and the local Ethics Committee at the University of Freiburg, Germany (no. 475/19) and was carried out in accordance with the Declaration of Helsinki. The CONSORT guidelines and their extension for randomized pilot and feasibility studies were followed for reporting this trial (http://www.consort-statement.org) (72, 74).

The study was conducted online in German and English language. Recruitment was carried out from March 2020 to February 2021 and took place mainly in Germany, with a small part also in Austria, Switzerland, and Liechtenstein. The last follow-up assessment was completed in July 2021. Emails, posts, and flyers referring to the study homepage were distributed through diverse recruitment channels. Among the channels were institutions working with refugees (e.g., universities, refugee support services, psychosocial centres for refugees), (social) media (newsletters, Twitter, Facebook), personal contacts and on-site advertisement (e.g., refugee accommodations). On the study homepage (https://esano.klips-ulm.de/de/trainings/schlafprobleme-sleep-problems/sleep-e/), interested participants could get detailed information on the study procedure, and they were provided a link to study registration.

After registering and signing the digital informed consent, participants were emailed a link to the baseline assessment (T1). Here, inclusion criteria for study participation were assessed, which were (1) age ≥ 18 years, (2) sufficient English or German language skills, (3) access to an internet device, (4) flight background. The only exclusion criterion was suicidality, unless the participant was in secured primary care. No cut-off for the severity of sleep disturbances was used as inclusion criterion so to obtain information on who might be interested in using the intervention, regardless of meeting clinical symptoms. This should also allow participation of refugees with subclinical symptoms to facilitate an initial and transdiagnostic access to mental healthcare. Eligible participants were randomized 1:1 to the IG or the waitlist CG after completing the baseline assessment. Randomization was conducted by an independent researcher not further involved in the study, using an automatic randomization software (https://www.sealedenvelope.com), applying permuted block randomization with randomly varying block sizes of two and four. Randomized participants were emailed information on the further study procedure. Participants of the IG received immediate access to the intervention after randomization; participants of the CG received access to the same intervention after completing the final assessment, following a waiting period with no intervention provided. Follow-up assessments took place 1 month (T2) and 3 months after randomization (T3). Participants received reminders to complete pending assessments. After T3, participants had the chance to win a 50€ Amazon voucher.

### Intervention

The used brief digital sleep intervention eSano Sleep-e was culturally adapted from GET.ON recovery, a cognitive behavioral digital intervention developed for German teachers showing large effects in reducing sleep disturbances (75–77). Adaptions were conducted between October 2017 and February 2020 and the procedure was based on the heuristic framework for the cultural adaptation of interventions [Barrera and Castro (60); illustrated in Supplementary Table 1.1]. Information on components to culturally adapt were gained in a literature review on culturally adapting digital interventions for mental disorders (64), and in a user experience study in which suggestions of inadequate intervention components were gathered in interviews with refugees and healthcare providers working with refugees (78). Adaptations included content adaptations (e.g., use relatable example characters with similar problems, expand psychoeducational elements, offer intervention in simplified German and English language) and methodological adaptations (e.g., use videos and images instead of text, provide intervention in a mobile format, shorten intervention). An overview on the components that have been culturally adapted is presented in Supplementary Table 1.2.

The intervention was accessible through the Minddistrict platform (https://www.minddistrict.com) in both a browser and mobile-based format. It consisted of four modules, which were recommended to be completed within 4 weeks. It took 30–45 min to complete all contents of one module; a module could be completed as a whole or broken down into different units. Contents aimed at normalizing mental health problems, promoting health behavior, and facilitating access to the health system. They included psychoeducation on sleep difficulties, rumination, and information on associated mental health conditions; sleep hygiene rules; exercises to deal with rumination; links to further mental healthcare options; and relaxation exercises (see Table 1). Information was mainly delivered via videos with experts and example characters and could be consolidated with exercises. Exemplary pages of the intervention are illustrated in Supplementary Table 2. The progress of each participant could be monitored on the platform, and when a module was completed, the next module was activated. In case a participant had not worked on the module for more than a week, they were reminded to do so via email, WhatsApp message, or phone call. No therapeutic guidance was provided.

**Table 1:** Contents of the four modules of the culturally adapted digital sleep intervention eSano Sleep-e.

**Module**	**Content**
Module 1	Introduction by a health expert, psychoeducation on sleep problems, reflecting on reasons to participate, relaxation exercise, offer to keep an online sleep diary
Module 2	Psychoeducation on sleep hygiene, exercise on sleep hygiene rules, psychoeducation on sleep medication, relaxation exercise, sleep diary
Module 3	Psychoeducation on rumination, exercises to deal with rumination, relaxation exercise, exercise on sleep hygiene rules, sleep diary
Module 4	Psychoeducation on problems related to sleep problems, information on mental healthcare options, reflecting on reasons to continue with exercises, relaxation exercise, reflecting on achievements, exercise on sleep hygiene rules, sleep diary

### Outcomes

Data was assessed via online self-report questionnaires and usage data from the intervention platform. At baseline, sociodemographic variables and medical and psychosocial treatments were assessed, and traumatic experiences as well as alcohol and drug consumption were screened with questions from the structured clinical interview for DSM-5 (79).

#### Adherence to the Intervention

Adherence to the intervention was evaluated by the mean (standard deviation, SD) of completed modules among the participants of the IG, as well as the rates of non-starters and completers. Data was derived from the intervention platform.

#### Acceptance of the Intervention

Acceptance outcomes included intervention satisfaction and its perceived cultural appropriateness. They were measured at T3 within those participants of the IG who had started the intervention at that time. Satisfaction with the intervention was assessed with the Client Satisfaction Questionnaire adapted for Internet Interventions (80) (CSQ-I; 8 items; scale 1–4; range 8–32, with a higher score indicating higher satisfaction; excellent McDonald's Ω = 0.95, excellent α = 0.95 in current sample). Perceived cultural appropriateness of the intervention was assessed with the self-developed Cultural Appropriateness Questionnaire, which was developed from a previous version (81) (CAQ; 21 items representing 4 subscales: structure, 7 items; design, 3 items; language, 2 items; content, 9 items; scale 1–5; total score range 21–105, with a higher score indicating higher perceived cultural appropriateness; acceptable α = 0.79 in current sample).

#### Effectiveness of the Intervention

The primary outcome was insomnia severity, measured by the Insomnia Severity Index (ISI) (82). Seven items are answered on a five-point Likert scale (0–4). The total score ranges from 0 to 28, with a higher score indicating higher insomnia severity. The internal consistency is excellent (Cronbach's α = 0.90); in the current sample, Cronbach's α was good (α = 0.84).

Several secondary outcomes were measured. Sleep quality was assessed with the Pittsburgh Sleep Quality Index (83) (PSQI; 19 items representing 7 components; scale 0–3; range 0–21, with a higher score indicating poorer sleep quality; good α = 0.83, questionable α = 0.63 in current sample). Fear of Sleep was assessed with the Fear of Sleep Inventory–Short Form (84) (FOSI-SF; 13 items; scale 0–4; range 0–52, with a higher score indicating higher fear of sleep; excellent α = 0.93, excellent α = 0.93 in current sample). Fatigue was assessed with the Multidimensional Fatigue Inventory (85) (MFI; 20 items representing 5 subscales with 4 items each: general fatigue, physical fatigue, reduced activity, reduced motivation, mental fatigue; scale 0–4; subscale score range 0–16, with a higher score indicating higher fatigue; good average α = 0.84, questionable α = 0.67 in current sample). Depressive symptoms were assessed with the Patient Health Questionnaire−9 item version (86) (PHQ-9; 9 items; scale 0–3; range 0–27, with a higher score indicating more severe depressive symptoms; good α = 0.89, good α = 0.88 in current sample). General wellbeing was assessed with the Refugee Health Screener−15, screening common mental disorders in refugees (posttraumatic stress disorder, anxiety, and depression) (87) (RHS-15; 14 items; scale 0–4; total score range 0–56, with a higher score indicating lower general wellbeing; excellent α = 0.92, excellent α = 0.91 in current sample). Mental health literacy was assessed with the Mental Health Literacy Questionnaire in young adults (88) (MHLQ; 29 items; scale 1–5; range 29–145, with a higher score indicating higher mental health literacy; good α = 0.84, good α = 0.87 in current sample). Literacy was assessed among four dimensions: knowledge on mental health problems, stereotypes, first aid skills, and self-help strategies.

#### Negative Effects of the Intervention

At T3, negative effects of the intervention were assessed within the IG with the Negative Effects Questionnaire, self-adapted to digital interventions (89, 90) [NEQ; 18 items; 3 scales per item: I. endorsement of a specific effect, scale 1 (yes), 0 (no); II. negativity of the effect, scale 0–4, with a higher score indicating a higher negativity; III. attribution of the effect, scale 1 (treatment), 0 (other circumstances)]. Negative effects represent five factors: symptoms, stigma, and hopelessness regarding mental health, dependency and perceived deficiencies regarding treatment.

### Statistical Analyses

Client satisfaction, perceived cultural appropriateness, adherence to the intervention, and negative effects of the intervention are illustrated descriptively.

Effectiveness analyses were performed using SPSS Statistics 27, and based on the intention-to-treat principle and on a two-side significance level of 5%. Intervention effects on the primary and secondary outcomes at T2 and T3 were investigated by means of linear multilevel analyses, with time (T1, T2, T3) on level 1 and individual participants on level 2 (random intercept, fixed slope; diagonal variance structure). This analytical procedure was chosen as it allows for the simultaneous analysis of time and group effects (91). Missing values were estimated in the multilevel analyses. The factors group (IG vs. CG) and time of assessment (T1, T2, T3) as well as their interaction were included into the models on the outcomes, with the interaction indicating group differences in the respective outcome over time. Post-hoc pairwise group comparisons were conducted at T2 and T3 separately. Standardized between-group effect sizes with their 95% confidence intervals (CI) were calculated (Hedges' g) for T2 and T3 by dividing the model-based estimated group mean difference by the corrected pooled SD (92). Due to the pilot character of the trial, *p*-values are only reported for analyses on the primary outcome. In addition to the intention-to-treat analyses, per protocol analyses were conducted analyzing the intervention effect on the primary outcome, including only those participants of the IG that had completed the main part of the intervention at the time of T3.

We conducted stepwise multiple regression analyses to examine whether acceptance of and adherence to the digital intervention predicted changes in the primary or secondary outcomes from T1 to T3. Perceived cultural appropriateness, client satisfaction, and number of completed modules at the time of T3 were used as predictors.

## Results

### Participants

**Figure 1:**
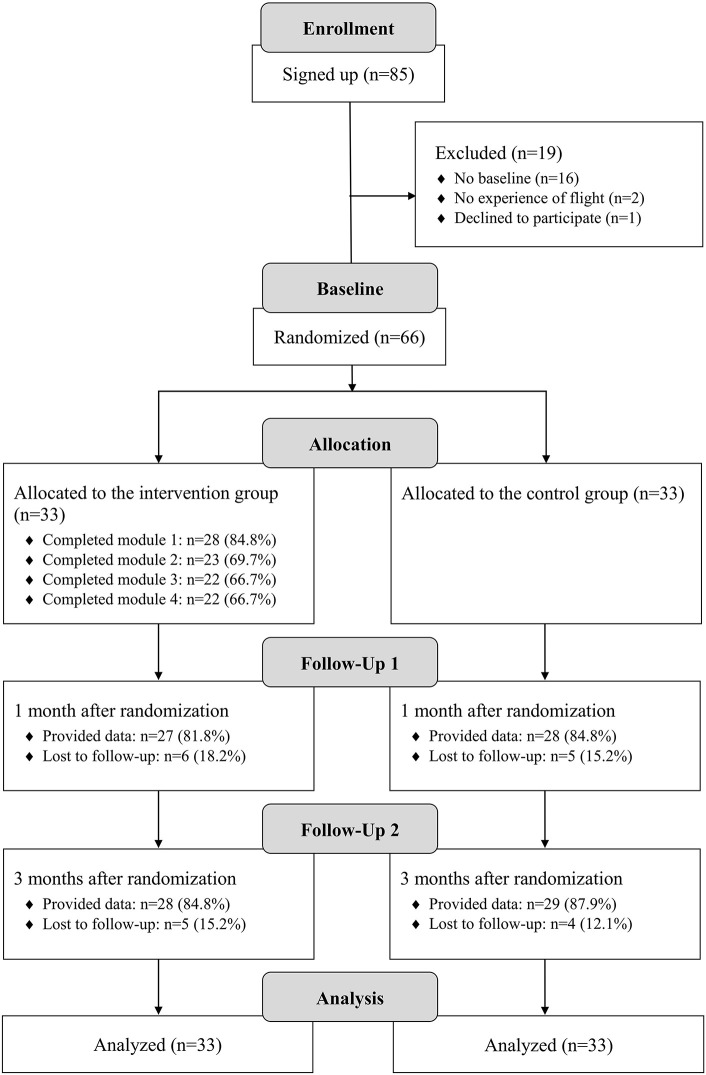
Study flow chart.

Figure 1 shows the participants' flow. Sixty-six participants were randomized to the IG (*n* = 33) and CG (*n* = 33). Their sociodemographic characteristics are illustrated in Table 1. Eighteen participants (27.3%) were female. The mean (SD) age was 28.5 (6.8) years, ranging from 18 to 49 years. Participants were from 14 nationalities, with the majority coming from Syria (*n* = 39, 59.1%). In total, 58 (87.9%) of the participants were from countries in the orient, the remaining 8 (12.1%) from sub-Saharan African countries. Participants spoke 15 different native languages, with 43 (65.2%) having Arabic as (one of) their native language. 36.4% of the participants (*n* = 24) were currently employed, 37.9% (*n* = 25) were enrolled at a university. Thirteen participants (19.7%) were in current psychotherapeutic treatment, 45 (68.2%) had never sought psychological help before. Regarding baseline symptomatic, three participants did not show clinically significant insomnia severity; 30 (45.5%) showed subclinical insomnia severity, and 33 (50.0%) showed clinical insomnia severity. Sixty-two participants (93.9%) reported past traumatic experiences, 14 (21.2%) reported alcohol or drug abuse.

Sociodemographic data (Table 2) and baseline symptoms (Tables 3, 4) did not differ substantially between the IG and CG, except for the number of participants being in therapeutic treatment [IG: 3 (9.1%); CG: 10 (30.3%)]. Neither did the study dropout rates differ between the groups [IG: 6 (18.2%), CG: 5 (15.2%) dropout at T3]. Differences between participants who completed T3 (*n* = 57) and those who dropped out of the study (*n* = 9), or between participants of the IG who completed the training (*n* = 22) and those who did not (*n* = 11) were revealed concerning language abilities, level of education, and student status. Rates of lower language skills and lower educational level were higher among participants who did not complete T3 or the intervention, whereas rates of higher language skills and higher educational level were enhanced among participants who completed T3 and the intervention. Similarly, the number of university students was higher among study and intervention completers than among non-completers. Detailed information on the revealed group differences are presented in Supplementary Table 3.

**Table 2:** Sociodemographic and baseline characteristics of study participants.

**Characteristic**	**All participants (***n*** = 66)**	**IG (***n*** = 33)**	**CG (***n*** = 33)**
Age in years, mean (SD)	28.5 (6.8)	29.2 (6.9)	27.7 (6.6)
Gender, female, *n* (%)	18 (27.3)	10 (30.3)	8 (24.2)
In a relationship, married, *n* (%)	26 (39.4)	9 (27.3)	17 (51.5)
**Educational level,** ***n*** **(%)**			
No qualification/ primary school	6 (9.1)	2 (6.1)	4 (12.1)
Middle school	11 (16.7)	6 (18.2)	5 (15.2)
High school/ university degree	49 (74.2)	25 (75.8)	24 (72.7)
**Employment,** ***n*** **(%)**			
Employed	24 (36.4)	13 (39.4)	11 (33.3)
Student	25 (37.9)	11 (33.3)	14 (42.4)
House wife/husband, unemployed	16 (24.2)	8 (24.2)	8 (24.2)
Years in Germany, mean (SD)	4.7 (1.5)	4.8 (1.5)	4.7 (1.6)
Language eSano Sleep-e, German, *n* (%)	56 (84.8)	30 (90.6)	26 (78.8)
**German/English language skills,** ***n*** **(%)^a^**			
Beginner, elementary	2 (3.0)	1 (3.0)	1 (3.0)
Intermediate, upper-intermediate	26 (39.4)	14 (42.4)	12 (36.5)
Pre-advanced, advanced	36 (54.6)	18 (54.5)	18 (54.5)
Current use of psychotherapy, *n* (%)	13 (19.7)	3 (9.1)	10 (30.3)
**Previous use of psychological help,** ***n*** **(%)**			
No	45 (68.2)	21 (63.3)	24 (72.2)
Internet/counseling center	8 (12.1)	5 (15.2)	3 (9.1)
Doctor/psychotherapist	17 (25.8)	9 (27.3)	8 (24.2)
Insomnia (ISI)^b^, *n* (%)			
Not clinical	3 (4.5)	3 (9.1)	0 (0.0)
Subthreshold	30 (45.5)	13 (39.4)	17 (51.5)
Clinical–moderate	27 (40.9)	15 (45.5)	12 (36.4)
Clinical–severe	6 (9.1)	2 (6.1)	4 (12.1)
Traumatic experience	62 (93.9)	31 (93.9)	31 (93.9)
Alcohol and drug abuse	14 (21.2)	6 (18.2)	8 (24.2)
	**All participants (*****n*** **= 66)**		
Birth country (*n*)	Syria (39), Afghanistan (6), Turkey (3), United Arab Emirates (3), Iraq (2), Nigeria (2), Gambia (2),		
	Sierra Leone (2), Libanon (2), Iran (1), Eritrea (1), Somalia (1), Egypt (1), Sudan (1)		
Native language (*n*)	Arabic (40), Kurdish (5), Kurdish + Arabic (3), Persian (3), Dari (2), Mandinka (2), Paschtu (2),		
	Turkish (2), Edo (1), Igbo (1), Krio (1), Kurdish + Turkish (1), Somali (1), Temne (1), Tigrinya (1)		

*IG, intervention group; CG, control group; SD, standard deviation; ISI, Insomnia Severity Index (82)*.

a *language skills according to the language of participation in the study (English/German)*;

b *ISI total score categories: 0–7, no clinically significant insomnia; 8–14, subthreshold insomnia; 15–21, clinical insomnia (moderate); 22–28, clinical insomnia (severe)*.

**Table 3:** Results for the primary outcome measured by the Insomnia Severity Index (82).

	**IG**		**CG**		**Adjusted effect estimates**			**Interaction**
	***N***	**Observed mean (SD)**	***N***	**Observed mean (SD)**	**Mean difference (95% CI)**	***p*** **-value**	**Hedges' g (95% CI)**	**(time x group)**
T1	33	14.7 (5.2)	33	15.2 (5.2)				*F*_2, 60_= 0.88, *p* = 0.421
T2	27	12.0 (6.5)	28	14.2 (5.0)	-2.1 (-4.8–0.5)	0.112	0.40 (-0.09–0.88)	
T3	28	11.5 (5.0)	29	12.9 (5.0)	-1.6 (-4.3–1.2)	0.257	0.28 (-0.20–0.77)	

*IG, intervention group; CG, control group; SD, standard deviation; CI, confidence interval; T1, Baseline assessment; T2, 1-month follow-up assessment; T3, 3-months follow-up assessment*.

**Table 4:** Results for the secondary outcomes.

	**IG**		**CG**		**Adjusted effect estimates**	
	**N**	**Observed mean (SD)**	***N***	**Observed mean (SD)**	**Mean difference (95% CI)**	**Hedge's g (95% CI)**
**Sleep quality (PSQI)**						
T1	33	9.3 (3.2)	33	10.0 (3.7)		
T2	27	7.9 (4.0)	28	9.2 (4.3)	-1.0 (-3.0–1.0)	0.26 (-0.23–0.74)
T3	28	6.8 (3.8)	29	8.9 (3.6)	-1.8 (-3.8–0.3)	0.42 (-0.07–0.91)
**Fear of sleep (FOSI-SF)**						
T1	33	10.0 (10.6)	33	10.1 (12.2)		
T2	27	7.7 (9.4)	28	8.0 (12.0)	-1.1 (-6.5–4.4)	0.10 (-0.39–0.58)
T3	28	7.4 (9.3)	29	6.4 (9.9)	0.3 (-5.0–5.6)	-0.03 (-0.51–0.46)
**Fatigue–general fatigue (MFI subscale 1)**						
T1	33	9.0 (3.7)	33	9.0 (3.1)		
T2	27	7.7 (4.0)	28	8.8 (3.5)	-1.0 (-2.8–0.8)	0.26 (-0.22–0.75)
T3	28	7.2 (3.7)	29	8.5 (3.1)	-1.5 (-3.3–0.3)	0.40 (-0.08–0.89)
**Fatigue-physical fatigue (MFI subscale 2)**						
T1	33	8.1 (3.8)	33	7.5 (3.5)		
T2	27	7.3 (4.0)	28	8.3 (4.6)	-0.9 (-2.9–1.2)	0.21 (-0.27–0.70)
T3	28	6.2 (4.0)	29	8.4 (3.3)	-2.1 (-4.0–0.2)	0.53 (0.04–1.02)
**Fatigue–reduced activity (MFI subscale 3)**						
T1	33	8.8 (4.0)	33	9.0 (4.8)		
T2	27	7.6 (4.3)	28	9.1 (4.0)	-1.2 (-3.2–0.9)	0.28 (-0.20–0.77)
T3	28	7.8 (4.2)	29	8.1 (4.0)	-0.6 (-2.9–1.8)	0.11 (-0.37–0.60)
**Fatigue–reduced motivation (MFI subscale 4)**						
T1	33	8.6 (3.6)	33	9.0 (3.9)		
T2	27	7.6 (3.9)	28	8.7 (4.7)	-1.1 (-3.3–1.0)	0.26 (-0.22–0.75)
T3	28	7.5 (4.3)	29	7.8 (4.1)	-0.6 (-2.7–1.5)	0.14 (-0.35–0.62)
**Fatigue–fatigue (MFI subscale 5)**						
T1	33	6.9 (3.5)	33	7.3 (3.0)		
T2	27	6.1 (3.2)	28	7.0 (3.9)	-0.7 (-2.5–1.1)	0.20 (-0.30–0.68)
T3	28	5.8 (3.7)	29	6.1 (3.1)	-0.4 (-2.1–1.3)	0.11 (-0.37–0.59)
**Depressive symptoms (PHQ-9)**						
T1	33	12.3 (6.2)	33	11.8 (6.4)		
T2	27	9.6 (6.7)	28	11.7 (6.1)	-2.0 (-5.0–1.0)	0.32 (-0.16–0.81)
T3	28	8.6 (6.4)	29	10.9 (5.9)	-2.3 (-5.7–1.0)	0.34 (-0.15–0.83)
**General wellbeing (RHS-15)**						
T1	33	20.9 (12.5)	33	21.1 (11.1)		
T2	27	18.1 (12.3)	28	20.5 (12.0)	-3.2 (-9.2–2.7)	0.27 (-0.22–0.75)
T3	28	17.1 (12.1)	29	20.4 (11.0)	-3.7 (-9.7–2.3)	0.30 (-0.19–0.79)
**Mental health literacy (MHLQ)**						
T1	33	113.5 (13.7)	33	115.5 (12.0)		
T2	27	115.3 (10.8)	28	115.9 (13.4)	-1.4 (-7.8–4.9)	0.11 (-0.37–0.59)
T3	28	115.5 (14.6)	29	113.7 (15.1)	0.0 (-7.8–7.7)	0.00 (-0.48–0.48)

*IG, intervention group; CG, control group; SD, standard deviation; CI, confidence interval; T1, Baseline assessment; T2, 1-month follow-up assessment; T3, 3-months follow-up assessment; PSQI, Pittsburgh Sleep Quality Index (83); FOSI-SF, Fear of Sleep Inventory-Short Form (84); MFI, Multidimensional Fatigue Inventory (85); PHQ-9, Patient Health Questionnaire 9 (86); RHS-15, Refugee Health Screener (87); MHLQ, Mental Health Literacy Questionnaire–young adults form (88)*.

### Adherence to the Intervention

Participants of the IG completed a mean of 2.9 (SD = 1.7) modules, which equals 72.0% of the intervention. Five participants (15.2%) did not start the intervention, 22 participants (66.7%) completed all modules (see Figure 1). At the time of T3, the mean of completed modules was 2.8 (SD = 1.7; 69.7%), with six participants (18.2%) not having started the intervention. Twenty participants (60.6%) had completed all modules at that time, and an additional two participants had completed three modules.

### Acceptance of the Intervention

Intervention satisfaction among the participants of the IG who started the intervention was found to be high with a mean CSQ-I score of M = 24.0 (SD = 6.6). The lowest rate of agreement was revealed in the statement “The training has met my needs,” which 15 participants (57.7%) partly or totally agreed with. The highest rate of agreement was shown in the statements “The training helped me deal with my problems more effectively,” “In an overall, general sense, I am satisfied with the training,” and “I would come back to such a training if I were to seek help again,” which 19 participants (71.4%) partly or totally agreed with, respectively. Details on the ratings are shown in Supplementary Table 4.

Perceived cultural appropriateness was shown to be high with a mean CAQ global score of M = 79.3 (SD = 10.3). All four subscales yielded high mean scores, with language being the scale with the lowest ratings (70.0% of total score) and structure being the scale with the highest ratings (77.4%). The lowest perceived appropriateness was shown on the item “It would have been better for me to do the training in my native language,” which 23.1% (strongly) supported. The highest appropriateness was shown on the item “I think the training looks good,” which 88.5% (strongly) supported. Details on the ratings are displayed in the Supplementary Table 5.

### Effectiveness of the Intervention

#### Primary Outcome

**Figure 2:**
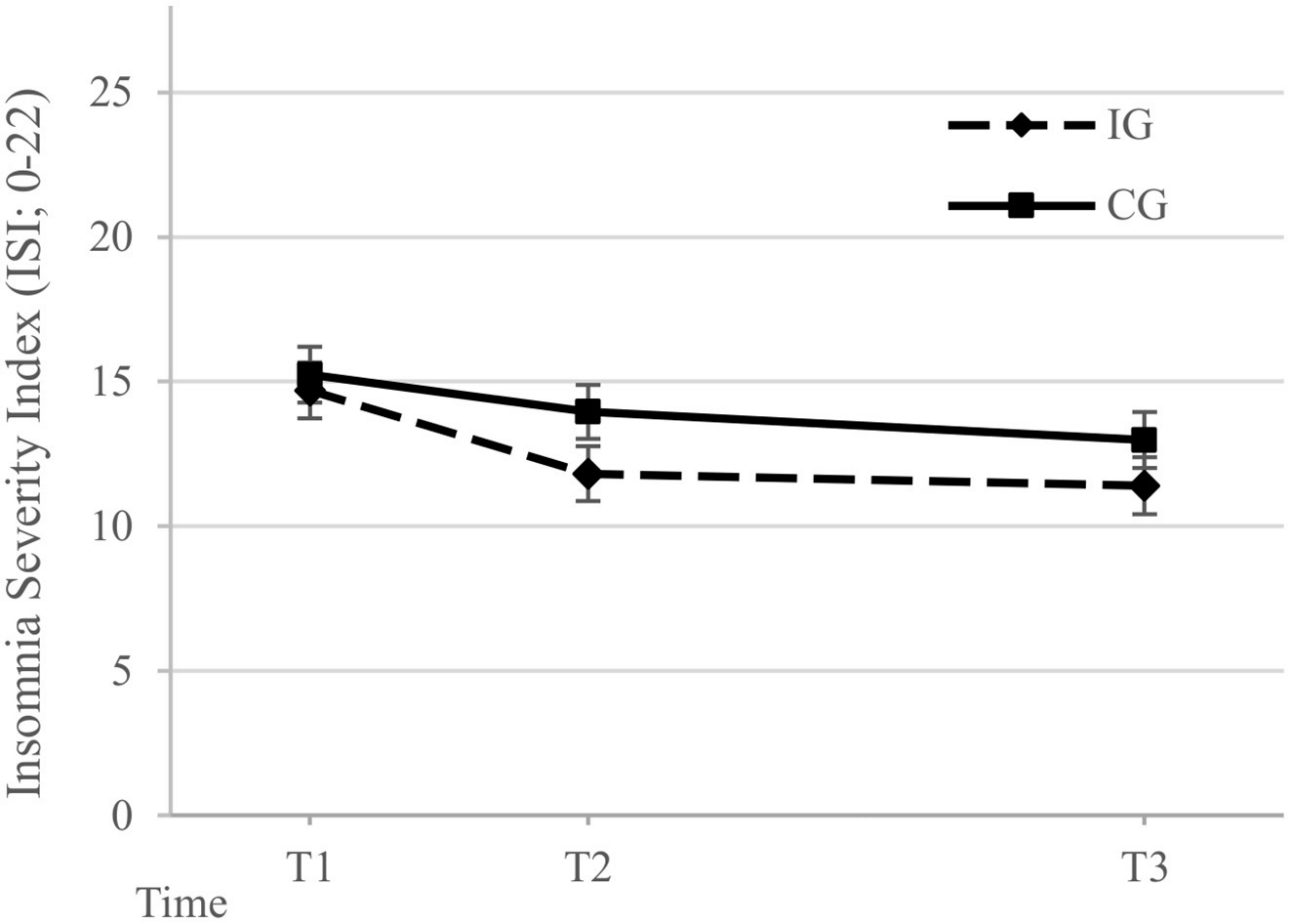
Model-based estimates of the Insomnia Severity Index scores (82) in the intervention group (IG) and control group (CG) at Baseline (T1), 1-month follow-up assessment (T2), and 3-months follow-up assessment (T3). The model includes the factors time, group, and their interaction. The error bars represent the estimated standard error of the mean.

Multilevel analyses of the ISI are illustrated in detail in Table 3, Figure 2. No significant group x time interaction was shown. At T1, the IG showed an insomnia severity of M = 14.7 (SD = 5.2) and the CG of M = 15.2 (SD = 5.2). At T2, a mean of M = 12.0 (SD = 6.5) in the IG and of M=14.2 (SD = 5.0) in the CG was observed, with a non-significant estimated mean difference of -2.1 (95% CI, -4.8 to 0.5, *p* = 0.112) and an effect size of g = 0.40. At T3, a mean of M = 11.5 (SD = 5.0) was observed in the IG and a mean of M = 12.9 (SD = 5.0) in the CG, with a non-significant estimated mean difference of -1.6 (95% CI, -4.3 to 1.2, *p* = 0.257) and an effect size of g = 0.28.

Per protocol analyses on the primary outcome were conducted including only those 22 participants of the IG that had completed at least three modules of the intervention at the time of T3. Analyses revealed a non-significant group x time interaction effect. Between-group mean differences of insomnia severity at T2 with -2.4 (95% CI, -5.2 to 0.4) and T3 with -1.9 (95% CI, -4.9 to 1.2) were non-significant, revealing effect sizes of g = 0.49 and g = 0.35, respectively. Detailed results are illustrated in Supplementary Table 6.

#### Secondary Outcomes

Table 4 presents all means and SD of the secondary outcomes. For the subscale physical fatigue of the MFI, a significant group x time interaction effect was shown with *F*_2, 54_= 4.97, *p* = 0.010, revealing a significant estimated mean difference at T3 between the IG and CG of -2.1 (95% CI, -4.0 to -0.2, *p* < 0.05; g = 0.53).

#### Prediction of Effectiveness of the Intervention

Regression analyses investigating the influence of the CSQ-I, the CAQ, and the number of completed modules at T3 on the primary outcome did not yield significant results. Among the secondary outcomes, scores of the PSQI, the FOSI, the PHQ, the RHS, and some subscales of the MFI were significantly predicted by CSQ-I or CAQ scores. None of the outcomes was significantly predicted by the adherence to the intervention. Results are illustrated in Supplementary Table 7.

### Negative Effects of the Intervention

All in all, 98 negative effects were reported that occurred during study participation (M = 3.8, SD = 3.5), of which 12 (12.2%) were reported to be caused by the intervention, and 86 (87.8%) were reported to be caused by other circumstances. Concerning the negative effects caused by the intervention, three related to the factor symptoms, with slightly, moderately, or strongly worsened symptoms; five related to the factor deficiencies, revealing slightly or moderately perceived deficiencies of the intervention; one related to the factor dependency, with a moderate dependency on the intervention; two related to the factor stigma, with a moderate or strong stigma in relation to the intervention; and one related to the factor hopelessness, with a moderate hopelessness due to the intervention. Details on negative effects are displayed in Supplementary Table 8.

## Discussion

This is the first trial investigating a digital sleep intervention in refugees. Thereby, three low-threshold approaches were combined to engage refugees: using a scalable digital format for the intervention, addressing somatically perceived and transdiagnostic sleep disturbances, and culturally adapting the intervention. The findings of this non-confirmatory trial indicate that the self-help intervention holds the potential to provide multiple burdened refugees with initial access to mental health treatment. So, on the one hand, although there were no inclusion criteria to the study regarding a cut-off for insomnia severity, only three of 66 participants showed no clinically significant insomnia severity based on the ISI (82). Moreover, 93.9% participants reported from previously endured traumata, 89.4% showed at least mild depressive symptoms, and 77.3% revealed current mental health problems. On the other hand, more than two-thirds (68.2%) of the participants never had used psychological help before.

The particular target group of burdened and underserved refugees showed high satisfaction with, perceived appropriateness of, and adherence to the digital sleep intervention, with rates comparable to the original intervention (77, 81). Importantly, the intervention did not seem to cause any serious negative effects. However, in this non-confirmatory pilot trial, intervention effects on insomnia severity were shown to be non-significant and small, with g = 0.40 after 1 month, and g = 0.28 after 3 months comparing the IG to the waitlist CG. Of note, the power to find significant insomnia severity differences was 1-β = 0.35 at T2 and 1-β = 0.20 at T3. Nevertheless, effects are smaller than the revealed large effects of the culturally non-adapted versions (77, 81). Effects on the secondary outcomes were also mostly small. A moderate effect was revealed only on reducing physical fatigue, which fits the assumed somatic mental health concept of refugees (93). High improvements in the CG might have leveled out between group differences, possibly due to an increased use of psychotherapeutic treatment among the CG (30.3% of the CG vs. 9.1% of the IG). Furthermore, the intervention was developed as a brief, psychoeducative health promotion intervention, not comprising recommended and effective components of sleep treatment that require high self-motivation and are demanding for users (e.g., sleep restriction and stimulus control) (94). This approach was chosen because the primary goal of the intervention was to provide refugees with initial access to healthcare, to be followed by more intensive treatment for sleep disturbances or other mental health conditions (37, 95, 96).

When interpreting the results, two aspects should be taken into account. First, a large proportion of participants had high levels of education and language skills, and the intervention dropout rate seemed higher among participants with lower levels of education and language skills. Second, almost three-quarters (72.7%) of the IG participants needed to be motivated to start or continue with the intervention at least three times, and intervention completion took long (average days of completion: 46, range 12 to 107). This may suggest that the intervention completion was difficult and required skills that could mainly be fulfilled among highly educated refugees, which may be an easier-to-reach subgroup of refugees.

To increase adherence, acceptance, and effectiveness in refugees with a lower level of education, it might be necessary to further adapt the intervention. (1) As suggested by the results of the CAQ, the intervention should be translated into native languages of refugees. (2) As access to the intervention was shown to be difficult by the frequent reminders on starting the intervention, the intervention should be provided on an easy-to-access platform with lower technical demands. In addition to reminders, persuasive design principles aiming at user engagement may be promising in this regard (97). (3) More guidance should be provided to support the refugees on technical or other demands, as guidance is known to improve intervention adherence and effectiveness (33, 98). After such a refinement of the cultural adaptation, its acceptance and effectiveness should be compared with the present version of the intervention in a sufficiently powered randomized controlled trial (63, 67, 99), or in randomized factorial trials (100, 101). These allow the modification of single intervention components (e.g., language modifications) and thereby the comparison of the specific effect of the suggested refinements of cultural adaptation to understand mechanisms of change more precisely (102, 103), and may thus be a fruitful next step in further developing the present intervention. In addition to a further adaptation of the intervention, the study conduction should be optimized: (1) Questionnaires that have been developed and evaluated for refugees and are available in their native languages ought to be applied (87); and (2), more in-person recruitment should be used, so that refugees with a lower level of education and technical familiarity can be reached (104).

The conducted pilot trial had several limitations. First, due to the pilot character of the trial, the sample size and, thus, the power to detect clinical significant effects was limited. Second, the digital sleep intervention and study participation material was only available in English or German. This posed high demands on (possible) participants, potentially hindering interested refugees with higher language barriers from participating in the study and the intervention. Third, along with this, a large part of the participants had a high level of education, which does not adequately reflect the situation of refugees, herewith indicating a selection bias and, thus, a limited generalisability. Fourth, a large proportion of Syrian refugees participated in the study. Although this roughly maps the distribution of refugees in Germany, the generalisability for refugees from other countries of origin is limited. Fifth, the used self-report questionnaires to assess the effectiveness and acceptability of the intervention were largely not evaluated for the target population, with exception of the RHS-15 (87). This lack of evaluation or possibly also of cultural appropriateness for refugees might be linked with the revealed questionable internal consistency of some questionnaires (PSQI; MFI). Sixth, no information on the participants' thoroughness in completing the intervention was gained; however, before activating the following module, it was verified that the main parts of a module had been visited. Seventh, we did not differentiate between refugees with regard to, for example, length of stay in Germany or country of origin. However, such aspects may influence the need of culturally adapting digital interventions and, herewith, the effectiveness of such interventions (105) and should thus be considered in a future randomized trial.

## Conclusions

In conclusion, this pilot trial indicated that providing a treatment in a digital format, targeting transdiagnostic sleep disturbances, and conducting a cultural adaptation resulted in an intervention that seemed feasible to establish initial healthcare access for hard-to-reach, burdened refugees. Acceptance and adherence levels were high, and no serious negative effects due to the intervention were reported. However, this non-confirmatory pilot trial indicated only a small, non-significant intervention effect on improving insomnia severity. Evaluating a refined cultural adaptation focusing on language, intervention complexity, and engagement strategies may be a promising next step toward bridging the mental health treatment gap for refugees.

## Data Availability Statement

The raw data supporting the conclusions of this article will be made available by the authors, without undue reservation.

## Ethics Statement

The studies involving human participants were reviewed and approved by Ethics Committee at the University of Freiburg, Germany. The patients/participants provided their written informed consent to participate in this study.

## Author Contributions

KSpa, JB, and LS conceived the study conceptualization and design. DL provided the intervention template GET.ON recovery, KSpa and LS adapted the intervention content, HB provided access to the intervention platform. KSpi provided expertise in sleep problems and treatment. KSpa and EH collected data, conducted the statistical analyses, and wrote the draft of the manuscript. HB, JB, and LS supervised the work. All authors contributed to the manuscript revision, read, and approved the submitted version.

## Funding

KSpa is supported by the German Academic Scholarship Foundation. The article processing charge was funded by the University of Freiburg in the funding programme Open Access Publishing.

## Conflict of Interest

The authors declare that the research was conducted in the absence of any commercial or financial relationships that could be construed as a potential conflict of interest.

## Publisher's Note

All claims expressed in this article are solely those of the authors and do not necessarily represent those of their affiliated organizations, or those of the publisher, the editors and the reviewers. Any product that may be evaluated in this article, or claim that may be made by its manufacturer, is not guaranteed or endorsed by the publisher.

## Footnotes

^1^This number includes refugees and asylum-seekers, further referred to as *refugees*.

## Abbreviations

CAQ: Cultural Appropriateness Questionnaire
CG: control group
CI: confidence interval
CSQ-I: Client Satisfaction Questionnaire adapted to Internet interventions
FOSI-SF: Fear of Sleep Inventory-Short Form
IG: intervention group
ISI: Insomnia Severity Index
MFI: Multidimensional Fatigue Inventory
MHLQ: Mental Health Literacy Questionnaire young adults form
NEQ: Negative Effects Questionnaire
PHQ: Patient Health Questionnaire
PSQI: Pittsburgh Sleep Quality Index
RHS: Refugee Health Screener
SD: standard deviation
UNHCR: United Nations High Commissioner for Refugees.
