# Supplementary material for: Engaging Refugees With a Culturally Adapted Digital Intervention to Improve Sleep: A Randomized Controlled Pilot Trial
Source: Front Psychiatry. 2022 Feb 23;13:832196. doi: 10.3389/fpsyt.2022.832196 (PMC8905517; doi:10.3389/fpsyt.2022.832196)
Supplement: Supplementary file 1 [file Data_Sheet_1.pdf]

Supplementary Material

Supplementary Table 1.1 Cultural adaptation process of the digital intervention eSano Sleep-e, based on the heuristic framework for the cultural adaptation of interventions by Barrera and Castro (2006). 2

Supplementary Table 1.2 Conducted cultural adaptations on the digital intervention eSano Sleep-e, systematised according to the Taxonomy of Cultural Adaptation of Digital Interventions (Spanhel et al., 2021). 3

Supplementary Table 2 Exemplary pages of the digital sleep intervention (mobile format).10

Supplementary Table 3.1 Relevant differences between completers and non-completers of the 3-months follow up (FU2) on sociodemographic characteristics assessed at baseline. 11

Supplementary Table 3.2 Relevant differences between completers and non-completers of the digital sleep intervention on sociodemographic characteristics assessed at baseline. 11

Supplementary Table 4 Satisfaction with the digital sleep intervention according to the Client Satisfaction Questionnaire adapted for Internet Interventions (Boß et al., 2016), evaluated by participants of the intervention group (n=26). 12

Supplementary Table 5.1 Perceived cultural appropriateness of the digital sleep intervention according to the self-developed Cultural Appropriateness Questionnaire, evaluated by participants of the intervention group (n=26). 13

Supplementary Table 5.2 Means (standard deviations) of the self-developed Cultural Appropriateness Questionnaire und subscales, evaluated by participants of the intervention group (n=26). 13

Supplementary Table 6 Results of the per protocol analyses for the primary outcome measured by the Insomnia Severity Index (Bastien et al., 2001). 14

Supplementary Table 7 Results for the prediction of the change in the effectiveness outcomes by acceptance and adherence outcomes among participants of the intervention group (n=26), analysed with stepwise regression models. 15

Supplementary Table 8 Negative effects reported by the participants of the intervention (n=26), assessed with the Negative Effects Questionnaire (Rozental, Kottorp, Boettcher, Andersson, & Carlbring, 2016). 16

Supplementary references 17

Supplementary Table 1.1 Cultural adaptation process of the digital intervention eSano Sleep-e, based on the heuristic framework for the cultural adaptation of interventions by Barrera and Castro (2006).

Procedure according to the framework	Procedure of the adaptation of eSano Sleep-e
**1)** Gather information on the adaptation	• Systematic literature review to inform on previously conducted cultural adaptations of digital mental health interventions (Spanhel et al., 2021).<br>• User experience study to inform on required adaptations of the original intervention by conducting interviews with refugees and healthcare providers (Spanhel et al., 2019).
**2)** Develop a preliminary adaptation design	Adaptations as shown in Supplementary Table 1.2 were conducted, based on step 1)
**3)** Test the preliminary adaptation	Randomised controlled pilot trial to test the first version of eSano Sleep-e, with the results presented in this manuscript.
**4)** Refine and evaluate the adaptation	Pending.

Supplementary Table 1.2 Conducted cultural adaptations on the digital intervention eSano Sleep-e, systematized according to the Taxonomy of Cultural Adaptation of Digital Interventions (Spanhel et al., 2021).

	Components & sub-components	Characteristics to consider	Resulting adaptations
**Content components**	**1. Illustrated characters**		
	Content/ stories / background of characters	Target group of people with a flight background and their specific stories	Introduced role models that illustrate characters with a flight background, representing the target group with diverse relatable stories and problems
	Appearances/ names of characters	Refugees coming from diverse home countries and regions; more male than female refugees, rather younger than older refugees	Role models with culturally diverse looks and names; two male, one female character; rather young characters
	Role of the characters	Mental health problems are taboo among refugees	Role of the characters was to normalize having sleep disturbances and seeking help for them by reporting on how the intervention helped them
	**2. Illustrated activities**		
	Daily life	Daily lives of refugees might differ, among others caused by not being allowed to work and having family in the home country	Everyday habits, activities, and jobs were changed to relevant ones, e.g., watch television, play on smartphone/ use it for speaking to people in home countries, listen to music was included
	Religious/ traditional activities	Religion and spirituality are important to refugees and part of their daily lives	Religious activities were included, e.g. to pray
	Coping strategies	Knowledge on helpful activities is often small among refugees	Strategies to deal with rumination were expanded, relaxation exercises were added in each session
	**3. Illustrated environment/ burdens**		
	Mental health	Mental health of refugees is influenced by many stressors	Different reasons for sleeping problems and other mental health problems were considered and valued/ normalized

Components & sub-components	Characteristics to consider	Resulting adaptations
Countries, politics	Specific environments of the living situation in the arrival countries	Specific recommendations were adapted to situation of refugees, e.g. *prepare bed to signalize* bedtime rather than *preparing bedroom nicely*, due to not having a separate and own bedroom
Education	Refugees often have a low level of education and a high level of illiteracy	Explanations and contents were simplified
Burdens	Pre-, peri- and post-migration stressors: e.g., traumata, war and flight experiences; worries on the own security or the security of family; poor living conditions)	Pictures that might be associated with the flight (e.g., the sea, boats) were changed to neutral pictures; role models had relatable experiences
**4. Illustrated values/ traditions**		
Value/ importance of family/ community	Family is important to refugees	The importance of becoming healthy again was emphasized based on the aim of being there for the family (reason to get health again)
Value/ importance of religion/ respect	Religion and spirituality are important to refugees	Religion was valued in the stories of the role models
**5. Language visualization**		
Use of verbal expressions (sayings, quotes)	Metaphors and sayings vary across different languages and are not understandable to everyone	Quotes, sayings, and puns were excluded
**6. Language translation**		
Translating intervention	Refugees may not have sufficient German language skills	Intervention was translated from German to English
Providing various language options to choose	Choosing the best language option might be helpful	Refugees could choose to do the intervention in German or English

Components & sub-components	Characteristics to consider	Resulting adaptations
**7. Language tailoring**		
Simplify/ shorten text	Refugees are not used to reading long texts and often have a low level of education	Language was simplified and concretized, technical/academic terms were avoided and changed for easier-to-understand expressions, e.g., *rumination* was changed to *bad thoughts that do not stop*
Use of milder descriptions for mental health terms	Mental disorders are often stigmatized or tabooed	Terms such as *posttraumatic stress disorder* or *depression* were changed to *thoughts about bad experiences* or *being sad*
**8. Concepts of mental health (treatment)**		
Related to religion/ supernatural powers/ spirituality	Traditional/ religious healing methods are rather used than western healthcare providers	Traditional concepts of mental disorders and their treatment were accepted, but western treatment was explained to provide an alternative model
Poor knowledge	Refugees often have small knowledge on mental health (treatment), and low experience with mental healthcare providers	Basic explanations on mental disorders and their treatment in Germany were provided
Stigmatization of mental health problems	Mental disorders and seeking help are often stigmatized or tabooed and, thus, not accepted	Mental disorders and treating them was normalized by illustrating the frequency in videos with experts/ role models
Handling of mental health problems	Refugees often have mental health concepts that differ from western concepts, include traditional healing, and a somatic expression of feeling; low expectation of self-efficacy	Information on the healthcare system, treatment possibilities, and mental disorders was added, somatic problems were included in psychoeducational elements
Distrust in treatment/ confidentiality	Refugees often do not trust western treatment methods or western healthcare providers	Privacy and data security issues were emphasized, especially before starting the intervention, as well as the shown effectiveness of such programs

Components & sub-components	Characteristics to consider	Resulting adaptations
**9. Goals of treatment**		
Increase understanding/ acceptance of mental disorders	Mental disorders are often stigmatized or tabooed	Psychoeducational elements on various mental disorders were increased; mental disorders were normalized by illustrating the frequency in videos with experts/ role models
Increase understanding on treatment possibilities	Refugees should be integrated into the healthcare systems in the arrival countries	Self-efficacy and an active approach of problems was promoted by, e.g. including information on adequate handling and quitting of medication; information on the healthcare system and treatment possibilities was added
Enhance coping strategies	Knowledge on helpful activities is often small among refugees	Focus on sleep hygiene rules and relaxation exercises was increased
**10. Methods of treatment**		
Comprehensive psychoeducation	Refugees often have mental health concepts that differ from western concepts	A lot of basic psychoeducation on sleep problems, mental health problems, and links of sleep and other mental health problems were provided by the experts; western treatment methods were explained by the experts to increase understanding and legitimation
Information/ links to other helpful addresses	Refugees often have small knowledge on mental healthcare systems	Links to places where to find help for mental health problems were provided
Emphasis on positive outcomes	Refugees often do not trust western treatment methods	Peoples' resources and goals were focused to increase positive expectancy; e.g., example characters had already overcome their sleep problems with the intervention
Other treatment methods	Relaxation exercises were perceived very well	Increased the part of relaxation exercises

	Components & sub-components	Characteristics to consider	Resulting adaptations
Methodological components	**11. Structure**		
	Shorten intervention	Refugees might not be used to long periods of concentration	Length of modules was shortened (4 shorter instead of 3 longer sessions were offered), and modules were structured in a way that enabled pauses in between
	Changes in texts	Refugees are not used to reading long texts	Text sequences were shortened, information was mainly presented via videos
	Simplify introduction	Information should be presented clearly and directly	Introductions and overview pages were shortened or deleted
	Add optional intervention elements	Obligations to do exercises is reluctantly received	All content is offered optional and can be done in whichever order (within one module), which mainly is important for the exercises (sleep diary, quiz, sleep hygiene rules, relaxation exercises)
	**12. Functionality**		
	Provide more explanations/ examples	Refugees are often not used to psychotherapeutic content	Exercises are explained or exemplified by experts or example characters, with exemplary completions illustrated
	Simplify navigation	Many refugees are not used to computer interactions and to reading long texts	Audio-visual elements were used rather than texts; a simple format was used to navigate through the module
	Include interactive elements	Refugees enjoyed relaxation exercises	More relaxation exercises were added
		Obligation or rectification of views is reluctantly received	Quiz questions were reduced; sleep diary was offered as an option (not as a mandatory part)
	Computer- versus Smartphone	Many refugees do not have computer access, but own a smartphone	A mobile version was created in addition to computer version
	Additional intervention modules	Refugees often have small knowledge on mental healthcare systems	Information and addresses on where to get help was added

Components & sub-components	Characteristics to consider	Resulting adaptations
**13. Design and aesthetics**		
Changes in videos/ pictures	Reading is difficult for many refugees, watching videos and pictures is easier to understand	Enhanced the amount of videos and pictures
	Refugees are a diverse group of people	Illustrated diverse people: e.g. different skin color, clothes
	Videos and pictures can evoke trauma associations	Excluded images and pictures linked to flight experiences, e.g. the sea/ boats
**14. Guidance**		
Format of guidance	Many refugees are in need of mental healthcare, which makes a scalable intervention important	Both a guided and unguided version previously existed, of which the unguided version was used; reminders to continue with the training were made by mail/ WhatsApp/ phone calls
Amount of guidance	Refugees often have less structured days, time and days are less important	The amount of reminders to continue with the training was enhanced to reach refugees

	Components & sub-components	Characteristics to consider	Resulting adaptations
Procedural components	**15. Methods used to obtain information**		
	Pilot/ feasibility study	Pilot randomized controlled trial to evaluate the culturally adapted digital intervention among refugees	
	Personal interaction	User experience study with qualitative interviews with healthcare providers and refugees	
	Literature review	Systematic review on previously conducted cultural adaptation of digital mental health interventions	
	**16. Persons involved**		
	Target group, associated people	Refugees from heterogeneous home countries	
	Professionals	Healthcare providers working with refugees	
	**17. Theoretical framework**		
	Guideline for cultural adaptation of face to face treatment	Barrera, M., & Castro, F. G. (2006). A heuristic framework for the cultural adaptation of interventions. Clinical Psychology: Science and Practice, 13(4), 311–316. https://doi.org/10.1111/j.1468-2850.2006.00043.x	
		Bernal, G., Bonilla, J., & Bellido, C. (1995). Ecological validity and cultural sensitivity for outcome research - issues for the cultural-adaptation and development of psychosocial treatments with hispanics. Journal of Abnormal Child Psychology, 23(1), 67–82. https://doi.org/10.1017/CBO9781107415324.004	
	Guideline for developing digital interventions	Baumel, A., Faber, K., Mathur, N., Kane, J. M., & Muench, F. (2017). Enlight: a comprehensive quality and therapeutic potential evaluation tool for mobile and web-based eHealth interventions. Journal of Medical Internet Research, 19(3), e82. https://doi.org/10.2196/jmir.7270	
		Kim, P., Eng, T. R., Deering, M. J., & Maxfield, A. (1999). Review of published criteria for evaluating health-related websites. Western Journal of Medicine, 170(6), 329–332.	
		Stoyanov, S. R., Hides, L., Kavanagh, D. J., Zelenko, O., Tjondronegoro, D., & Mani, M. (2015). Mobile App Rating Scale: a new tool for assessing the quality of health mobile apps. JMIR mHealth and uHealth, 3(1), e27. https://doi.org/10.2196/mhealth.3422	

Note: Cultural adaptation based on a) a conducted user experience study with health experts and refugees (Spanhel et al., 2019), and b) a conducted systematic review on previously conducted cultural adaptations on digital interventions (Spanhel et al., 2021).

Supplementary Table 2 Exemplary pages of the digital sleep intervention (mobile format).

Introducing the sleep diary, module 1

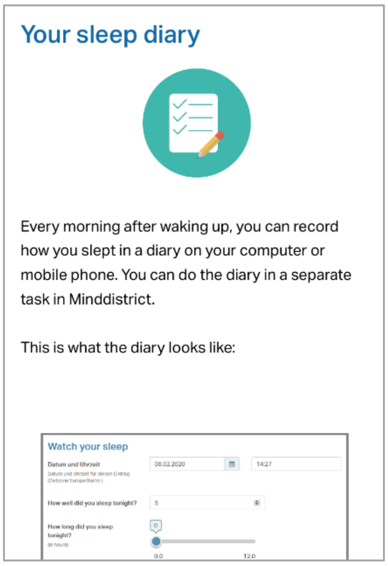

Overview on module content, module 2

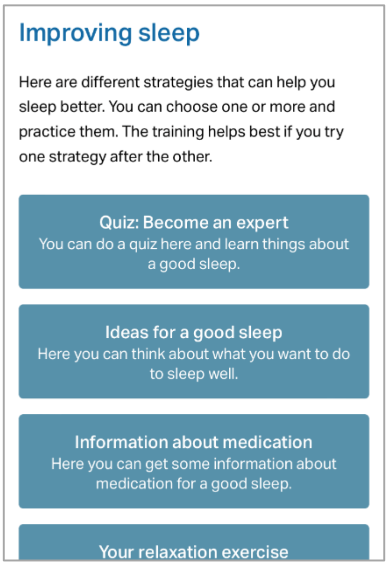

Quiz on sleep / sleep hygiene, module 2

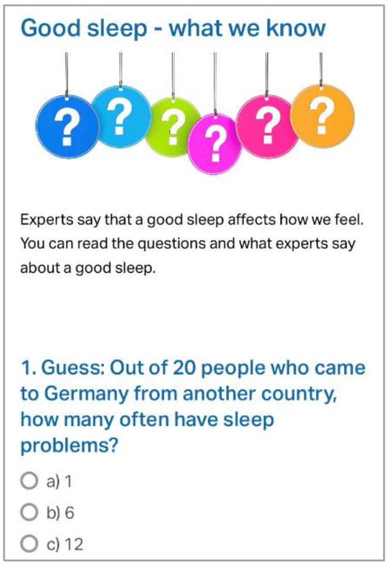

Psychoeducation on rumination, module 3

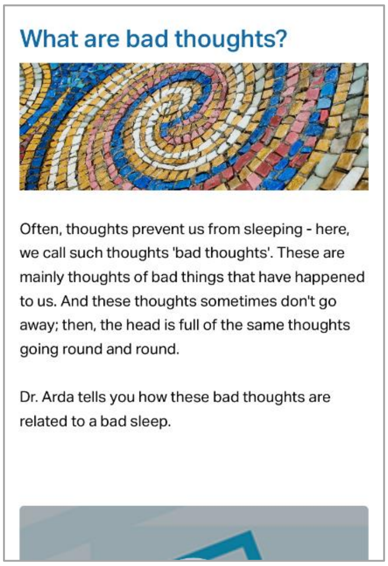

Exercise on rumination, module 3

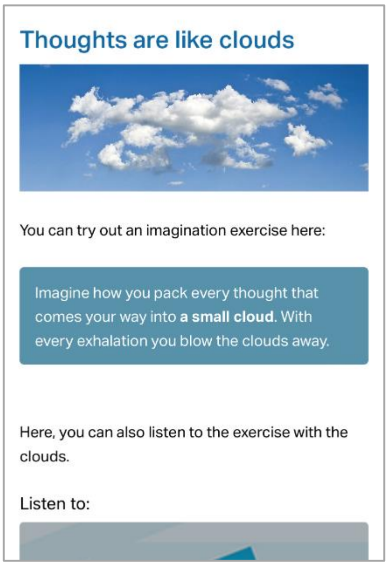

Outlook, module 4

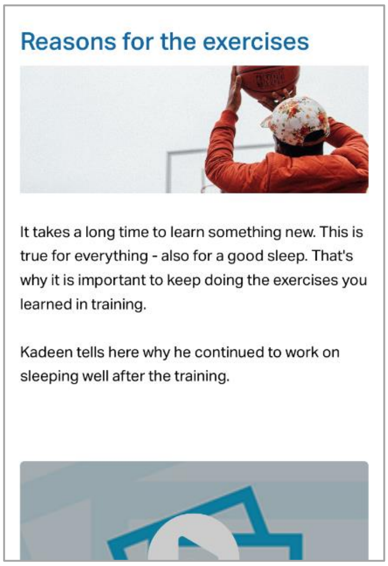

Supplementary Table 3.1 Relevant differences between completers and non-completers of the 3-months follow up (FU2) on sociodemographic characteristics assessed at baseline.

	**FU2 completer (n=57)**	**FU2 non-completer (n=9)**
Educational level, n (%)		
No qualification/ primary school	4 (7.0)	2 (22.2)
Middle school	8 (14.0)	3 (33.3)
High school/ university degree	45 (78.9)	4 (44.4)
German/English language skills, n (%)[a]		
Beginner, elementary	1 (1.8)	1 (11.1)
Intermediate, upper-intermediate	20 (36.4)	6 (66.7)
Pre-advanced, advanced	34 (61.8)	2 (22.2)
Student status, n (%)	23 (40.4)	2 (22.2)

[a] FU2 completer: n=55

Supplementary Table 3.2 Relevant differences between completers and non-completers of the digital sleep intervention on sociodemographic characteristics assessed at baseline.

	**Intervention group – completer (n=22)**	**Intervention group – non-completer (n=11)**	**Control group (n=33)**
Educational level, n (%)			
No qualification/ primary school	1 (4.5)	1 (9.1)	4 (12.1)
Middle school	2 (9.1)	4 (36.4)	5 (15.2)
High school/ university degree	19 (86.4)	6 (54.5)	24 (72.7)
German/English language skills, n (%)[a]			
Beginner, elementary	1 (4.5)	0 (0)	1 (3.2)
Intermediate, upper-intermediate	6 (27.3)	8 (72.7)	12 (38.7)
Pre-advanced, advanced	15 (68.2)	3 (27.3)	18 (58.1)
Student status, n (%)	9 (36.0)	2 (18.2)	14 (42.4)

[a] Control group: n=31

Supplementary Table 4 Satisfaction with the digital sleep intervention according to the Client Satisfaction Questionnaire adapted for Internet Interventions (Boß et al., 2016), evaluated by participants of the intervention group (n=26).

Satisfaction ratings	N (%)
The quality of the training rated as partly or totally high.	18 (69.2)
Partly or totally received the kind of training they wanted.	17 (65.4)
The training has partly or totally met their needs.	15 (57.7)
Would partly or totally recommend the training to a friend who needed similar help.	18 (69.2)
Partly or totally satisfied with the amount of help they received through the training.	18 (69.2)
The training partly or totally helped to deal with their problems more effectively.	19 (71.4)
Partly or totally satisfied with the training in general.	19 (71.4)
Would partly or totally come back to such a training if they were to seek help again.	19 (71.4)

Note: Analyses included participants of the intervention group who completed the 3-months follow-up assessment and had started with the intervention at the time of assessment.

Supplementary Table 5.1 Perceived cultural appropriateness of the digital sleep intervention according to the self-developed Cultural Appropriateness Questionnaire, evaluated by participants of the intervention group (n=26).

**Perceived cultural appropriateness ratings**	**N (%)** [a]
The training helped them.	21 (80.8)
They think the training looks good.	23 (88.5)
For them, the number of modules (four) was good.	22 (84.6)
They found it hard to find their way through the modules. [b]	17 (65.4)
They took breaks within the modules. [b]	7 (26.9)
They liked the pictures used in the training.	17 (65.4)
They found the colors of the training good.	18 (69.2)
The language used in the training was hard to understand. [b]	22 (84.6)
It would have been better for them to do the training in their native language. [b]	6 (23.1)
The content of the training was easy to understand.	20 (76.9)
The content of the training conflicted with their culture. [b]	20 (76.9)
They liked the relaxation exercises.	19 (73.1)
The information of the experts Prof. Kizilhan and Dr. Arda helped them.	21 (80.8)
The stories and explanations of Mariam, Kadeen, and Ali helped them.	18 (69.2)
They felt represented by Mariam, Kadeen, and Ali and their stories.	11 (42.3)
It was hard for them to integrate the exercises in their everyday life. [b]	13 (50.0)
They did some of the exercises several times.	14 (53.8)
They liked the number of videos in the training.	22 (84.6)
They liked the number of pictures in the training.	18 (69.2)
They liked the amount of text in the training.	22 (84.6)
They liked the number of audio exercises in the training.	21 (80.8)

Note: Analyses included participants of the intervention group who completed the 3-months follow-up assessment and had started with the intervention at the time of assessment.

[a] Number (percentage) of participants who rated the respective item with "agree" or "strongly agree".
[b] Inverted items: Number (percentage) of participants who rated the respective item with "disagree" or "strongly disagree".

Supplementary Table 5.2 Means (standard deviations) of the self-developed Cultural Appropriateness Questionnaire und subscales, evaluated by participants of the intervention group (n=26).

	**Mean (standard deviation)**
**Global score (max. 105)** [a]	**79.3 (10.3)**
Structure (max. 35)	27.1 (4.5)
Design (max. 15)	11.5 (2.3)
Language (max. 10)	7.0 (1.7)
Content (max. 45)	33.8 (5.2)

Note: Analyses included participants of the intervention group who completed the 3-months follow-up assessment and had started with the intervention at the time of assessment.

[a] Higher values express a higher perceived cultural appropriateness.

Supplementary Table 6 Results of the per protocol analyses for the primary outcome measured by the Insomnia Severity Index (Bastien et al., 2001).

	IG		CG		Adjusted effect estimates			Interaction (time x group)
	**N**	**Observed mean (SD)**	**N**	**Observed mean (SD)**	**Mean difference (95% CI)**	**p value**	**Hedges' g (95% CI)**	
**T1**	22	14.4 (5.6)	33	15.2 (5.2)				$F_{2,55}$= 0.72, p=.491
**T2**	22	11.5 (6.1)	28	14.2 (5.0)	-2.4 (-5.2 to 0.4)	.090	0.49 (-0.07 to 1.06)	
**T3**	22	11.1 (5.0)	29	12.9 (5.0)	-1.9 (-4.9 to 1.2)	.225	0.35 (-0.20 to 0.91)	

Note: Analyses included only participants of the intervention group who had completed ≥3 modules at the time of the 3-months follow-up assessment.

IG = intervention group; CG = control group; SD = standard deviation; CI = confidence interval; T1 = Baseline assessment; T2 = 1-month follow-up assessment; T3 = 3-months follow-up assessment.

Supplementary Table 7 Results for the prediction of the change in the effectiveness outcomes by acceptance and adherence outcomes among participants of the intervention group (n=26), analyzed with stepwise regression models.

Effectiveness outcomes	Acceptance/ adherence	B (SE)	β	T	p	95% CI
Insomnia severity (ISI)		no predictors entered				
Sleep quality (PSQI)		R^2 = .301; F(1,25) = 10.75				
	CSQ-I	0.29 (0.09)	.548	3.28	.003	0.11 to 0.47
	CAQ					
	Modules completed					
Fear of sleep (FOSI-SF)		R^2 = .156; F(1,25) = 4.61				
	CSQ-I	0.36 (0.17)	.394	2.15	.042	0.01 to 0.70
	CAQ					
	Modules completed					
Fatigue–general fatigue		R^2=.261; F(1,25) = 8.85				
(MFI subscale 1)	CSQ-I					
	CAQ	0.20 (0.07)	.511	2.97	.006	0.06 to 0.33
	Modules completed					
Fatigue–physical fatigue		R^2 = .147; F(1,25) = 4.29				
(MFI subscale 2)	CSQ-I					
	CAQ	0.12 (0.06)	.383	2.07	.049	0.00 to 0.23
	Modules completed					
Fatigue–reduced activity		R^2 = .171; F(1,25) = 5.16				
(MFI subscale 3)	CSQ-I					
	CAQ	0.16 (0.07)	.414	2.27	.032	0.02 to 0.31
	Modules completed					
Fatigue–reduced motivation		R^2 = .294; F(1,25) = 10.40				
(MFI subscale 4)	CSQ-I					
	CAQ	0.19 (0.06)	.542	3.23	.003	0.07 to 0.31
	Modules completed					
Fatigue–mental fatigue (MFI subscale 5)		no predictors entered				
Depressive symptoms (PHQ-9)		R^2 = .189; F(1,25) = 5.82				
	CSQ-I	0.37 (0.15)	.434	2.41	.024	0.05 to 0.68
	CAQ					
	Modules completed					
General well-being (RHS-15)		R^2 = .156; F(1,25) = 4.61				
	CSQ-I	0.58 (0.27)	.395	2.15	.042	0.02 to 1.14
	CAQ					
	Modules completed					
Mental health literacy (MHLQ)		no predictors entered				

Note: Analyses included participants of the intervention group who completed the 3-months follow-up assessment and had started with the intervention at the time of assessment. For participants with missing values, list-wise deletion was applied.

SE, standard error of the mean; CI, confidence interval; ISI, Insomnia Severity Index (Bastien, Valliéres, & Morin, 2001); PSQI, Pittsburgh Sleep Quality Index (Buysse, Reynolds, Monk, Berman, & Kupfer, 1989); FOSI-SF, Fear of Sleep Inventory-Short Form (Pruiksma et al., 2014); MFI, Multidimensional Fatigue Inventory (Smets, Garssen, Bonke, & De Haes, 1995); PHQ-9, Patient Health Questionnaire-9 items version (Kroenke, Spitzer, & Williams, 2001); RHS-15, Refugee Health Screener-15 items version (Hollifield et al., 2013); MHLQ, Mental Health Literacy Questionnaire-young adults form (Dias, Campos, Almeida, & Palha, 2018); CSQ-I, Client Satisfaction Questionnaire adapted for Internet Interventions (Boß et al., 2016); CAQ, Cultural Appropriateness Questionnaire (self-developed).

Supplementary Table 8 Negative effects reported by the participants of the intervention (n=26), assessed with the Negative Effects Questionnaire (Rozental, Kottorp, Boettcher, Andersson, & Carlbring, 2016).

	**Extremely (BI)**	**Very (BI)**	**Moderately (BI)**	**Slightly (BI)**	**Not at all (BI)**
I had more problems with my sleep.		4 (-)	3 (-)	1 (-)	
I felt like I was under more stress.	2 (-)	3 (-)	4 (-)	5 (1)	
I experienced more anxiety.	1 (-)		2 (-)	3 (-)	
I felt more worried.	1 (-)	1 (-)	4 (-)	3 (-)	
I experienced more hopelessness.		4 (-)	4 (-)		
I experienced more unpleasant feelings.		4 (-)	4 (1)	1 (-)	
I felt that the issue I was looking for help with got worse.		1 (-)	1 (-)	1 (-)	
Unpleasant memories resurfaced.	1 (-)	2 (1)	1 (-)	4 (1)	
I became afraid that other people would find out about my treatment.			1 (1)		
I got thoughts that it would be better if I did not exist anymore and that I should take my own life.		1 (-)	3 (-)	3 (-)	
I started feeling ashamed in front of other people because I was having treatment.		1 (1)		1 (-)	
I stopped thinking that things could get better.	1 (-)	1 (-)	3 (1)	1 (-)	
I started thinking that the issue I was seeking help for could not be made any better.		1 (-)	1 (-)	1 (-)	
I think that I have developed a dependency on my treatment.			4 (1)	1 (-)	
I did not always understand my treatment.		1 (-)		4 (1)	
I did not have confidence in my treatment.				1 (1)	
I felt that the treatment did not produce any results.			1 (1)	2 (1)	
I felt that the treatment was not motivating.					

Note: Analyses included participants of the intervention group who completed the 3-months follow-up assessment and had started with the intervention at the time of assessment.
BI = caused by intervention.

Supplementary references

Barrera, M., & Castro, F. G. (2006). A heuristic framework for the cultural adaptation of interventions. *Clinical Psychology: Science and Practice*, *13*(4), 311–316. https://doi.org/10.1111/j.1468-2850.2006.00043.x

Bastien, C. H., Valliéres, A., & Morin, C. M. (2001). Validation of the Insomnia Severity Index as an outcome measure for insomnia research. *Sleep Medicine*, *2*(4), 297–307. https://doi.org/10.1016/S1389-9457(00)00065-4

Boß, L., Lehr, D., Reis, D., Vis, C., Riper, H., Berking, M., & Ebert, D. D. (2016). Reliability and validity of assessing user satisfaction with web-based health interventions. *Journal of Medical Internet Research*, *18*(8), e234. https://doi.org/10.2196/jmir.5952

Buysse, D. J., Reynolds, C. F., Monk, T. H., Berman, S. R., & Kupfer, D. J. (1989). The Pittsburgh Sleep Quality Index: a new instrument for psychiatric practice and research. *Psychiatry Research*, *28*(2), 193–213. https://doi.org/10.1016/0165-1781(89)90047-4

Dias, P., Campos, L., Almeida, H., & Palha, F. (2018). Mental health literacy in young adults: adaptation and psychometric properties of the Mental Health Literacy questionnaire. *International Journal of Environmental Research and Public Health*, *15*(7), 1318–1330. https://doi.org/10.3390/ijerph15071318

Hollifield, M., Verbillis-Kolp, S., Farmer, B., Toolson, E. C., Woldehaimanot, T., Yamazaki, J., … SooHoo, J. (2013). The Refugee Health Screener-15 (RHS-15): Development and validation of an instrument for anxiety, depression, and PTSD in refugees. *General Hospital Psychiatry*, *35*(2), 202–209. https://doi.org/10.1016/j.genhosppsych.2012.12.002

Kroenke, K., Spitzer, R. L., & Williams, J. B. W. (2001). The PHQ-9: Validity of a brief depression severity measure. *Journal of General Internal Medicine*, *16*(9), 606–613. https://doi.org/10.1046/j.1525-1497.2001.016009606.x

Pruiksma, K. E., Taylor, D. J., Ruggero, C., Boals, A., Davis, J. L., Cranston, C., … Zayfert, C. (2014). A psychometric study of the Fear of Sleep Inventory-Short Form (FoSI-SF). *Journal of Clinical Sleep Medicine*, *10*(5), 551–558. https://doi.org/10.5664/jcsm.3710

Rozental, A., Kottorp, A., Boettcher, J., Andersson, G., & Carlbring, P. (2016). Negative effects of psychological treatments: an exploratory factor analysis of the Negative Effects Questionnaire for monitoring and reporting adverse and unwanted events. *PLoS ONE*, *11*(6), e0157503. https://doi.org/10.1371/journal.pone.0157503

Smets, E. M. A., Garssen, B., Bonke, B., & De Haes, J. C. J. M. (1995). The Multidimensional Fatigue Inventory (MFI) psychometric qualities of an instrument to assess fatigue. *Journal of Psychosomatic Research*, *39*(3), 315–325. https://doi.org/10.1016/0022-3999(94)00125-O

Spanhel, K., Balci, S., Feldhahn, F., Bengel, J., Baumeister, H., & Sander, L. B. (2021). Cultural adaptation of internet- and mobile-based interventions for mental disorders: a systematic review. *Npj Digital Medicine*, *4*, 128. https://doi.org/10.1038/s41746-021-00498-1

Spanhel, K., Schweizer, J. S., Wirsching, D., Lehr, D., Baumeister, H., Bengel, J., & Sander, L. (2019). Cultural adaptation of internet interventions for refugees: results from a user experience study in Germany. *Internet Interventions*, *18*, 100252. https://doi.org/10.1016/j.invent.2019.100252
